# Prevalence and Risk Factors of ACO (Asthma-COPD Overlap) in Aboriginal People

**DOI:** 10.1155/2018/4657420

**Published:** 2018-11-21

**Authors:** Adetola Koleade, Jamie Farrell, Gerald Mugford, Zhiwei Gao

**Affiliations:** ^1^Clinical Epidemiology Unit, Memorial University, Newfoundland and Labrador, Canada; ^2^Faculty of Medicine, Health Sciences Centre (Respirology Department), Memorial University, St John's, Newfoundland and Labrador, Canada

## Abstract

**Background and Objective:**

Aboriginal peoples are at a higher risk of many chronic respiratory diseases compared to the general Canadian population. Patients with asthma-COPD overlap (ACO), a disease newly described in 2015, are associated with frequent exacerbations, rapid decline in lung function, poor quality of life, high mortality, and disproportionate utilization of health-care resources than patients with asthma and COPD alone. The objective was to investigate the prevalence and risk factors of ACO in Aboriginal peoples.

**Methods:**

Data from the 2012 Aboriginal Peoples Survey (APS) were used for this study. The ACO definition was based on the respondent giving positive responses to both of the following questions *“Do you/Does (name) have Asthma diagnosed by a health professional?”* and *“Do you/Does (name) have chronic bronchitis, emphysema or chronic pulmonary obstructive disease or COPD diagnosed by a health professional?” Results*. Aboriginal peoples older than 45 years, women, widowed, separated, or divorced, having a total personal income below $20,000 were associated with a significant risk of ACO. Residing in Ontario, being a daily smoker, living in a rented dwelling, dwelling in need of major repairs, having diabetes, and working more than 40 hrs a week were also significantly associated with increased risk of ACO.

**Conclusion:**

The results from this study will provide information to aid the development of prevention and intervention strategies for Aboriginal communities.

## 1. Introduction

Aboriginal peoples are at a higher risk of many chronic respiratory diseases compared to the general Canadian population [1–3]. A recent Canadian survey showed that approximately 15% of Aboriginal peoples had been diagnosed with at least one of the chronic respiratory diseases (chronic obstructive pulmonary disease (COPD), chronic bronchitis (CB), emphysema, and asthma) compared to 10% for non-Aboriginal peoples in Canada [4]. Inequalities in health status often result from social, cultural, economic, environmental, and political factors. Education level, occupation, income, rurality, accessibility to health care, and possible interplays between these determinants of health can lead to disparities. A higher prevalence of chronic respiratory diseases in Aboriginal peoples has been associated with many factors including higher smoking rate, poor housing, poor schooling, low household income, and lack of timely access to health care [5].

Obstructive airway diseases including asthma and COPD have been associated with social, economic, and health impact on individuals, families, and society in general [6]. In a US study, the prevalence of adult asthma was reported to be 7.7% in those aged 35–64 years, while the prevalence of COPD was between 6.6% and 9.2% across the age group of 45 to 64 years, and even higher from 11.6% to 12.1% across age 65 years and older [7, 8]. Recently, a new obstructive airway disease, the asthma-COPD overlap (ACO) was described, with its first treatment and management guidelines reported in 2015 [9]. However, little information is currently available including the prevalence of ACO and its associated risk factors. A recent study from Finland suggested that the prevalence of ACO was about 27% in asthma patients with a smoking history [10]. Another study suggested that about 10 to 20% of patients with COPD may have ACO [11].

Patients with ACO experience a greater health burden including worse respiratory symptoms, poorer health-related quality of life (QOL), frequent exacerbations leading to more emergency visits, comorbidities, and higher doses of medications, as compared to asthma and COPD alone [6, 12–14]. Given that Aboriginal peoples are at a higher risk of chronic respiratory diseases [1, 2, 15], there is a need to study the prevalence and risk factors of this new disease (ACO) in Aboriginal people. Data from the 2012 Aboriginal Peoples Survey (APS), a national survey with detailed information on the demographic, environmental, health, and lifestyle status of Aboriginal peoples provided a unique platform to address these questions.

## 2. Methods

### 2.1. Study Design

Data from the 2012 APS collected by Statistics Canada from February to July 2012 were used for this study. This is a national cross-sectional survey of First Nations living off reserve, Metis and Inuit. It collected detailed information on Aboriginal identity, education, culture, income, health status, housing, and family background. Respondents were chosen based on self-identification as being Aboriginal or having Aboriginal ancestry from the 2011 National Health Survey (NHS). This study included only Aboriginal peoples aged ≥12 years from whom the information on the diagnosis of COPD was collected.

### 2.2. Outcome Variable and Risk Factors

The primary outcome variable ACO was based on the respondent giving positive responses to both of the following questions *“Do you/Does (name) have Asthma diagnosed by a health professional?”* and *“Do you/Does (name) have chronic bronchitis, emphysema, or chronic pulmonary obstructive disease or COPD diagnosed by a health professional?”*

The variables of interest were categorized into Demographic, Environmental, Socioeconomic, and Lifestyle variables and other diseases. Demographic variables consist of *Age, Sex,* and *Marital Status*. Environmental variables consist of *Rural or Urban (This is defined by the NHS Population Centre size); Province; Dwelling—owned or rented; Dwelling*—*need repairs; and Number of people in a household/Number of rooms in a dwelling*. Socioeconomic variables consisted of *Total Personal Income and Employment—the number of paid hours per week.* Lifestyle variables consisted of *Smoking Status and Anybody smoking in the dwelling* and other diseases such *as Diabetes*.

### 2.3. Statistical Analysis

Mean (standard deviation) and count (frequency) were calculated for continuous and categorical variables, respectively. Sampling weights were included in all statistical analyses. PROC SURVEYLOGISTIC was used to identify the significant risk factors for ACO in the univariate and multivariate analysis. Only clinically important factors and variables with a *p*-value lower than 0.20 in the univariate analysis were included in the multivariate analysis. To account for complex survey design of the APS, variances were estimated using 1,000 bootstrap weights with a Fay adjustment factor of 0.75. The level of significance *α* = 0.05 was used for the multivariate logistic regression. Data analysis was conducted using SAS version 9.4.

## 3. Results

### 3.1. Descriptive Statistics

The distribution of the population is shown in Table 1. The prevalence of ACO in the aboriginal population was 2.7%.

Examinations of demographic variables showed that almost half the population was between the age of *12 to 34 years* (45%) followed by those aged 3*5 to 44 years* (17%), *45 to 54 years* (18%), *55 to 64 years* (12%), and *65 years and over* (8%). Fifty-four percent of the sample were *Women*. *Married and Living in common-law* represented the highest proportion of 48% followed closely by *Single and never married* (38%) while the *Widowed, separated, and divorced group* was 14%.

**Figure 1:**
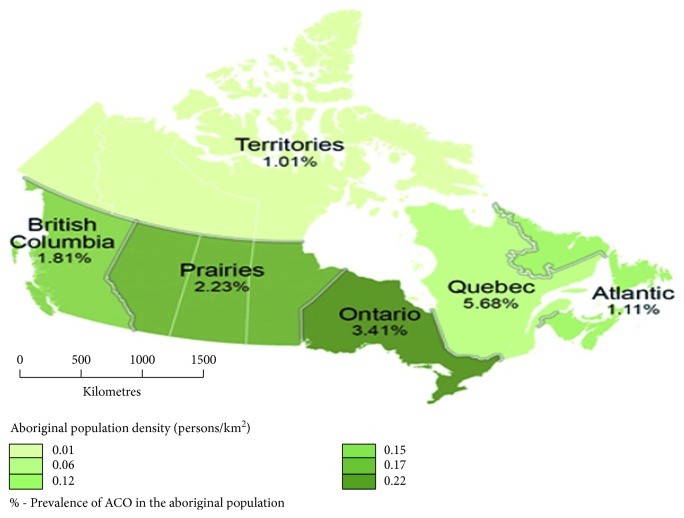
Map showing the Aboriginal population density and prevalence of ACO geographically in Canada.

Examination of environmental variables showed that individuals from a *Large urban population centre* (100,000 or more) represented the highest proportion (43%), followed by *Rural area,* which was 24%, and *Small population centre* (1,000 to 29,999) was 21% while the *Medium population centre* (30,000 to 99,999) had the least at 12%. Aboriginal people residing in the *Prairies (Alberta, Manitoba, Saskatchewan)* had the highest proportion (36%), followed by *Ontario* (25%), *British Columbia* (17%), *Quebec* (10%), *Atlantic Canada (Nova Scotia, Newfoundland and Labrador, Prince Edward Island, and New Brunswick)* (8%) while the *Territories (Nunavut, Yukon, Northwest Territories)* had the lowest (4%) (Figure 1). *Dwellings that needed only regular maintenance* recorded the highest proportion (62%) while *dwellings that required major repairs* yielded a proportion of (12%). Most people living in a dwelling of *3 to 5 rooms* yielded 45%, *6 to 8 rooms* recorded 33%, *9 rooms and over* recorded 16% while the least proportion was *0 to 2 rooms* with 6%.

Examination of socioeconomic status showed that individuals who earn *$20,000 to 49,999* per year had the highest proportion (32%), followed by those that earn between *$5,000 to 19,999* (28%). Individuals earning *$50,000 to $100,000 and over* were 23% while the lowest proportion was *$5,000 or less* with 18%. About employment hours per week, individuals working *80 hours and above* recorded the highest proportion (46%), *21 to 40 hours* yielded 37%, and *41 to 79 hours* per week and working part-time of *0 to 20 hours* yielded 9% and 8%, respectively. Individuals that *Owned a dwelling* had a higher proportion (58%) when compared to those that *Rent a dwelling* (42%).

Examination of lifestyle variables showed that individuals that smoked *Daily* had a proportion of 28% while those that smoked *Occasionally* recorded a lower proportion (9%). Individuals that *Smoke at home* recorded a proportion of 64% when compared to those that *do not smoke at home* (36%). Diabetes (type 1 and 2) was reported to be 9% of the respondents.

### 3.2. Univariate Analysis

The results from the Univariate analysis are shown in Table 2. *Age* was significantly associated with ACO. In comparison to those aged *12-34 years*, individuals who were older than 45 years were about three times more likely to have ACO. *Women* were two times more likely to be associated with ACO than *Men*. In comparison to those *Married or Living in common-law*, individuals who were *Widowed, Separated, and Divorced* and *Single and never married* were more likely to be associated with higher risks of ACO.

Individuals from the *Small population centre* were significantly less likely to be associated with ACO in comparison to individuals from a *Large urban population centre*. In comparison to Ontario, other provinces and regions were significantly less likely to be associated with ACO except for *Quebec*. Individuals residing in a *dwelling in need of major repairs* were three times more likely to be associated with ACO compared to those that reside in a *dwelling that needs only regular maintenance*. In comparison to those living in a dwelling of *0 to 2 rooms,* individuals living in a dwelling with *6*–*8 rooms* and *9 rooms and over* were significantly less likely to be associated with ACO.

Among the socioeconomic variables, individuals who earn between *$5,000 or less* to *$100,000 and over* were significantly less likely to be associated with ACO in comparison to individuals who earn *$5,000 to 19,999*. Individuals who worked *80 hours and over* were approximately four times more likely to be associated with increased risk for ACO when compared to *0 to 20 hours* of paid hours per week. Also, individuals *living in the rented dwelling* were three times more likely to be associated with ACO when compared to those *owning the dwelling*.

Among lifestyle variables, *Daily smoking* was more than two times more likely to be associated with ACO in comparison to individuals reporting *No smoking at all*. Furthermore, individuals with a report of *smoking at home* were two times more likely to be associated with ACO when compared to those with report *Not smoking at home*. Individuals who report a diagnosis of *Diabetes type 1 and 2* were three times more likely to be associated with ACO compared to those without the *diagnosis of diabetes*.

### 3.3. Multivariate Analysis

As shown in Table 3, the results from the multivariate analysis showed the following demographic variables were significantly associated with ACO: individuals aged between 45 and 54 years were two times more likely to be associated with ACO in comparison to individuals aged between *12 and 34 years*. *Women* were approximately two times more likely to be associated with ACO compared to *Men.* Also, individuals who were *widowed, separated, or divorced* were two times more likely to be associated with ACO compared to individuals who were either *married or living in common-law*.

In comparison to individuals from *Ontario*, those from *Atlantic regions*, *Territories*, and *British Columbia* were significantly less likely to be associated with ACO. Also, individuals living in a *dwelling in need of major repairs* were two times more likely to be associated with ACO compared to those living in a *dwelling* in *need of regular maintenance*.

Among the socioeconomic variables, the following three variables were significantly associated with ACO: Individuals who earn between *$5,000 and $19,999* were three times more likely to be associated with ACO compared to those who earn *$50,000 to $100,000 and over*. Individuals working for long hours of *41 to 80 hours* and *81 hours and over* were significantly associated with ACO compared to those working *0 to 20 hours per week*. Also, individuals who live in a *rented dwelling* were approximately two times more likely to be associated with ACO than those *owning a dwelling*.

Smoking was significantly associated with ACO: individuals who *smoke daily* were found to be about two times more likely to be associated with ACO compared to those that *do not smoke at all*. Aboriginal people with diabetes (type 1 and 2) were also approximately two times more likely to develop ACO compared to those without the diagnosis of diabetes.

## 4. Discussion

Using the APS dataset, our results suggest that Aboriginal peoples older than 45 years, women, widowed, separated, or divorced having a total personal income below $20,000 were associated with a significant risk of ACO. Residing in Ontario, being a daily smoker, living in a rented dwelling, dwelling in need of major repairs, having diabetes, and working more than 40 hrs a week were also significantly associated with increased risk of ACO.

Individuals aged 45 to 54 years old are two times more likely to be associated with ACO when compared to the younger individuals aged 12 to 34 years. In a longitudinal population-based study in the Netherlands, the authors reported that the risk of being diagnosed with COPD increased with age. A man who was free of COPD at age 40 had an increased risk of being diagnosed with COPD from 0.8% to 12% with increasing years from 10 to 40 years; while a woman of the same age had increased risk from 0.8% to 8.3% [16]. In another population-based cohort study from Ontario, Canada, estimating trends in the prevalence and incidence of concurrent physician-diagnosed asthma and COPD, the authors reported that the standardized prevalence increased by 10.5% from 2002 to 2012 mainly in young adults [17]. Additionally, a cross-sectional study among Aboriginal people assessed the risk factors associated with COPD. It was reported that individuals aged 55 and older were significantly associated with the risk of COPD [18]. We could find no other studies that focused on Aboriginal peoples with ACO.

Chronic respiratory diseases, especially COPD have always been attributed to men older than 40 years. However, recent findings suggest that there is a growing increase in women diagnosed with COPD. In a study of 1,633 residents from Saskatchewan, Canada, it was reported that in women, the combined effect of grain farming and smoking history had a significant association with CB but not in men [19]. Another study assessing the prevalence of CB in Aboriginal peoples reported that women had a higher prevalence than men [4]. Additionally, women with more severe COPD have a higher risk of hospitalization and death due to respiratory failure and possible comorbidities when compared to men [20]. Our study that appears to be the first to assess the risk of ACO in Aboriginal peoples suggests that Aboriginal women are approximately two times more likely to report ACO compared to men.

The association between obstructive airway diseases and marital status has been examined in many population studies. In a study, patients diagnosed with COPD were described and compared based on their nutritional status, gender, pulmonary function, and marital status. The authors reported that individuals diagnosed with COPD who lived alone had a worse nutritional status [21]. A longitudinal study in the US focused on the psychological imbalance caused by bereavement and divorce in relation to COPD. It was reported that remarriage after bereavement or divorce was associated with a significantly decreased risk of COPD onset [22]. Our study showed that Widowed, Separated, and Divorced Aboriginal peoples were found to be two times more likely to be associated with ACO compared to those married or living common-law.

We reported significant geographic variation in the prevalence of ACO with people in Ontario being at a significantly higher risk of ACO compared to people from other provinces or regions. A study in Ontario, Canada, assessed individuals with asthma and COPD to see if higher levels of exposure to air pollution will increase the risk for ACO [23]. The authors reported that individuals exposed to higher levels of air pollution had nearly three times the risk of developing ACO [23]. The same group of researchers in a longitudinal cohort of women reported that the risk of COPD increased by more than 20% with each unit increase in exposure to PM_2.5_ [24].

In our study, we reported that Aboriginal peoples living in dwellings in need of major repairs were two times more likely to develop ACO. A study examining the differences in hospitalization for respiratory tract infections among First Nations using the 2006 census reported that poor housing conditions and income were contributing factors in hospitalization [25]. Another study from Saskatchewan, Canada, which assessed the prevalence of CB in two Aboriginal communities, reported that houses with a musty smell of mould were positively associated with CB [26]. This is also consistent with a study from the United States, which reported that 15% of people who reported a musty smell in their dwelling also reported CB and asthma [27].

Meanwhile, studies have shown that lower socioeconomic status is associated with respiratory diseases [28, 29]. Total personal income and paid employment hours in our study suggested Aboriginal peoples working over 40 hours a week and earning a low annual income of $20,000 were more likely to develop ACO when compared to Aboriginal peoples earning an income of $50,000 or greater and working same or fewer hours. A large population-based study of 8,028 individuals reported that low income and low quality of education were risk factors for asthma and COPD [29]. In a cross-sectional study that focused on the associated factors of COPD among Aboriginal peoples, the authors reported that Aboriginal peoples making less than the median income of $20,600 were at a higher risk to be associated with COPD [18].

There is still conflicting information about the impact of work hours on chronic respiratory diseases. A longitudinal study that continued for a 32-year period made use of the National Longitudinal Survey of Youth 1979. It collected information on job histories and work hours in relation to chronic disease status. The authors reported that there were no significant findings for an association between long hours and asthma [30]. This was not consistent with the result of our study. This could be due to the homogeneity of the Aboriginal population used in our study compared to the general population used in this longitudinal study. In a study that focused on housing conditions, it was reported that homeownership was related to home quality [31]. Poorly maintained houses could also lead to the loss of vapour barrier, which allows areas of dampness that are prone to contamination with mould [32]. Owned dwellings tend to have their repairs fixed quicker than rented dwellings. Our results suggest that individuals renting a dwelling are also approximately three times more likely to develop ACO when compared to owning a house.

In our study, individuals who smoke daily were found to be about two times more likely to be associated with ACO compared to those that do not smoke. Even though cigarette smoking has decreased considerably over the past decades, there is still a significant link between positive smoking or the exposure to environmental tobacco and respiratory diseases [10, 33–35]. Aboriginal peoples are observed to have higher smoking rates compared to the general Canadian population [5], but there are not many studies that have focused on the association between smoking and ACO. Kiljander et al. investigated the prevalence of ACO among 190 asthmatic patients with a smoking history. These patients had no previous diagnosis of COPD but were either current or ex-smokers with a history of at least ten pack years. It was reported that 27% of the patients were found to have ACO [10]. Another study from Sweden examining the association between environmental tobacco smoke (ETS) and risk of COPD showed that ETS was independently associated with COPD. However, the association was more significant with increased ETS exposure either at home, previous or current work, or at the three mentioned locations [33]. In two Aboriginal studies that investigated the factors associated with the prevalence of CB and COPD, daily or current smokers were significant compared to never smokers [4, 18].

In our study, Aboriginal peoples with a diagnosis of diabetes (either type 1 or type 2) were approximately two times more likely to develop ACO. Epidemiological studies have consistently reported that many socioeconomic and lifestyle factors such as smoking are significantly associated with both diabetes and chronic respiratory diseases [36, 37]. Pleasants et al. made use of the Behavioural Risk Factor Surveillance System (BRFSS) to assess the relationships between COPD, asthma, and comorbidities such as diabetes. It was reported that adults with overlap syndrome had the highest prevalence of diabetes [37].

In addition to the shared risk factors between chronic respiratory diseases and diabetes, the current medication for patients with asthma and COPD may also play a role. However, the results from different studies are not consistent. A nested case-control study from Quebec, Canada, assessed whether the use and dose of inhaled corticosteroids increase the risk of diabetes onset and progression in patients treated for respiratory diseases. It was reported that current use of inhaled corticosteroids was associated with a 34% increase in diabetes onset and progression while risks were even more significant at higher doses for the treatment of COPD [38]. Another study from Poland reported that concomitant diseases were diagnosed in 85% of patients with ACO, with the prevalence of diabetes being approximately 20% [39]. In contrast, a retrospective study evaluated whether there was an increased risk of new onset of diabetes or hyperglycemia among patients with asthma or COPD treated with inhaled corticosteroids. It was reported that treatment with inhaled corticosteroids in patients with asthma and COPD was not associated with increased risk of diabetes or hyperglycemia [40].

### 4.1. Limitations

There were several limitations to this study. The APS is a cross-sectional survey in which the information collected was gathered at a one-time period. This could lead to self-reporting bias or misclassification. Individuals self-reported the presence of asthma and COPD, which lacks clinical accuracy. All other answers in this survey were also self-reported, which could underestimate the prevalence of some variables.

## 5. Conclusion

To our knowledge, this is the first study to evaluate the prevalence and risk factors associated with ACO among Aboriginal peoples. Our study highlights the increasing prevalence of respiratory diseases in Aboriginal women. Even though ACO is a relatively new disease, our study still highlights the significance of smoking and dwelling in a house in need of major repairs, factors already known to be linked with respiratory diseases. Our study also highlights the association between ACO and concomitant diseases such as diabetes in Aboriginal peoples.

There is a need to better understand the burden and risk factors of ACO in Aboriginal peoples. The findings from this study will provide information to health-care workers, patients and their families, Indigenous governments/organizations, and government agencies.

## Figures and Tables

**Table 1:** Descriptive statistics for the variables in our study.

Variables	Labels	% of the population
Age	12 to 34	45
	35 to 44	17
	45 to 54	18
	55 to 64	12
	65 and over	8

Sex	Men	46
	Women	54

Marital status	Married and living common-law	48
	Widowed, separated, and divorced	14
	Single, never married	38

Rural or urban	Rural area	24
	Small population centre	21
	Medium population centre	12
	Large urban population centre	43

Personal income	$5000 or less income	18
	$5000 to $19,999	28
	$20,000 to 49,999	32
	$50,000 to $100,000 and over	23

Province	Atlantic^*∗*^	8
	Quebec	10
	Ontario	25
	Prairies	36
	British Columbia	17
	Territories^*∗∗*^	4

Smoking status	Daily	28
	Occasionally	9
	Not at all	63

Anybody smoking at home	Yes	64
	No	36

Dwelling (owned/rented)	Owned	58
	Rented	42

Dwelling in need of major repairs	Yes, major repairs are needed	12
	Yes, minor repairs are needed	26
	No, only regular maintenance is needed	62

Diabetes	Diabetes type 1 and type 2	9
	Gestational and no diagnosis of diabetes	91

How many rooms are there in a dwelling	0 and 2 rooms	6
	3 and 5 rooms	45
	6 and 8 rooms	33
	9 rooms and over	16

Number of paid hours per week	0 to 20 hours	8
	21 to 40 hours	37
	41 to 79 hours	9
	80 hours and over	46

^*∗*^Including Nova Scotia, Newfoundland and Labrador, Prince Edward Island, and New Brunswick. ^*∗∗*^Including Nunavut, Yukon, and Northwest Territories.

**Table 2:** Univariate analysis of the risk factors associated with the prevalence of ACO.

Variables	Prevalence of ACO	Odds ratio (95% CI)	*p* value
Age			
** **12 to 34	1.57	1	
** **35 to 44	1.68	1.07 (0.62–1.84)	0.8045
** **45 to 54	4.31	2.81 (1.71–4.61)	<0.0001
** **55 to 64	4.28	2.80 (1.71–4.59)	<0.0001
** **65 and over	4.88	3.20 (1.89–5.43)	<0.0001
Sex			
** **Men	1.65	1	
** **Women	3.53	2.18 (1.58–3.00)	<0.0001
Marital status			
** **Married and living common-law	1.87	1	
** **Widowed, separated, and divorced	6.76	3.80 (2.48–5.84)	<0.0001
** **Single, never married	2.64	1.42 (1.01–2.00)	<0.0001
Rural or urban			
** **Large urban population centre	3.00	1	
** **Rural area	2.17	0.72 (0.48–1.06)	0.0953
** **Small population centre	2.00	0.66 (0.45–0.98)	0.0370
** **Medium population centre	3.63	1.22 (0.74–2.00)	0.4346
Personal income			
** **$5000 to $19,999	5.85	1	
** **$5000 or less income	2.12	0.35 (0.22–0.55)	<0.0001
** **$20,000 to 49,999	2.18	0.36 (0.25–0.53)	<0.0001
** **$50,000 to $100,000 and over	0.81	0.13 (0.07–0.24)	<0.0001
Province			
** **Ontario	3.41	1	
** **Atlantic^*∗*^	1.11	0.32 (0.18–0.59)	0.0002
** **Quebec	5.68	1.72 (1.05–2.81)	0.0320
** **Prairies	2.23	0.65 (0.42–1.00)	0.0471
** **British Columbia	1.81	0.52 (0.32–0.86)	0.0100
** **Territories^*∗∗*^	1.01	0.29 (0.17–0.51)	<0.0001
Type of smoker			
** **Not at all	1.89	1	
** **Daily	4.47	2.42 (1.78–3.32)	<0.0001
** **Occasionally	2.38	1.26 (0.72–2.20)	0.4112
Anybody smoking at home			
** **No	2.38	1	
** **Yes	4.21	2.04 (1.42–2.94)	0.0001
Dwelling (owned/rented)			
** **Owned	1.58	1	
** **Rented	4.12	2.69 (1.95–3.70)	<0.0001
Dwelling in need of major repairs			
** **No, only regular maintenance is needed	2.01	1	
** **Yes, major repairs are needed	6.44	3.35 (2.19–5.13)	<0.0001
** **Yes, minor repairs are needed	2.47	1.24 (0.86–1.78)	0.2525
Diabetes			
** **Gestational and no diagnosis of diabetes	2.24	1	
** **Diabetes type 1 and type 2	7.20	3.38 (2.34–4.90)	<0.0001
How many rooms are there in a dwelling			
** **0 and 2 rooms	5.58	1	
** **3 and 5 rooms	3.70	0.65 (0.38–1.11)	0.1136
** **6 and 8 rooms	1.87	0.32 (0.16–0.63)	0.0010
** **9 rooms and over	1.23	0.21 (0.10–0.44)	<0.0001
Number of paid hours per week			
** **0 to 20 hours	1.19	1	
** **21 to 40 hours	1.22	1.03 (0.50–2.11)	0.9402
** **41 to 80 hours	2.09	1.77 (0.75–4.17)	0.1887
** **80 hours and over	4.21	3.65 (1.82–7.32)	0.0003

^*∗*^Including Nova Scotia, Newfoundland and Labrador, Prince Edward Island, and New Brunswick. ^*∗∗*^Including Nunavut, Yukon, and Northwest Territories.

**Table 3:** Multivariate analysis of the risk factors associated with the prevalence of ACO.

Variables	Labels	Odds ratio (95% CI)	*p* value
Age	12 to 34	1	
	35 to 44	1.01 (0.54 to 1.87)	0.9858
	45 to 54	2.43 (1.34 to 4.42)	0.0035
	55 to 64	2.00 (0.97 to 4.09)	0.0597
	65 and over	1.68 (0.71 to 3.99)	0.2406

Sex	Men	1	
	Women	1.74 (1.25 to 2.45)	0.0013

Marital status	Married and living common-law	1	
	Widowed, separated, and divorced	1.97 (1.19 to 3.25)	0.0080
	Single, never married	1.44 (0.94 to 2.20)	0.0908

Personal income	$50,000 to $100,000 and over	1	
	$5000 or less income	1.59 (0.71 to 3.54)	0.2559
	$5000 to $19,999	3.00 (1.44 to 6.23)	0.0033
	$20,000 to 49,999	1.80 (0.89 to 3.66)	0.1019

Province	Ontario	1	
	Atlantic^*∗*^	0.31 (0.16 to 0.61)	0.0007
	Quebec	1.58 (0.94 to 2.64)	0.0834
	Prairies	0.73 (0.47 to1.14)	0.1660
	British Columbia	0.51 (0.30 to 0.88)	0.0158
	Territories^*∗∗*^	0.21 (0.12 to 0.39)	<0.0001

Type of smoker	Not at all	1	
	Daily	1.66 (1.14 to 2.41)	0.0084
	Occasionally	1.10 (0.52 to 2.00)	0.9896

Dwelling (owned/rented)	Owned	1	
	Rented	1.76 (1.24 to 2.51)	0.0018

Dwelling in need of major repairs	No, only regular maintenance is needed	1	
	Yes, major repairs are needed	2.31 (1.46 to 3.65)	0.0004
	Yes, minor repairs are needed	1.15 (0.79 to 1.69)	0.4579

Diabetes	Gestational and no diagnosis of diabetes	1	
	Diabetes type 1 and type 2	1.68 (1.10 to 2.58)	0.0188

Number of paid hours per week	0 to 20 hours	1	
	21 to 40 hours	1.16 (0.55 to 2.44)	0.7004
	41 to 80 hours	2.83 (1.12 to 7.14)	0.0273
	80 hours and over	2.85 (1.36 to 5.97)	0.0057

^*∗*^Including Nova Scotia, Newfoundland and Labrador, Prince Edward Island, and New Brunswick. ^*∗∗*^Including Nunavut, Yukon, and Northwest Territories.

## Data Availability

The 2012 Aboriginal Peoples Survey used to support the findings of this study has not been made available because it is not a publicly available dataset. It is available upon request from Statistics Canada.
